# Ethical challenges nurses faced during the COVID-19 pandemic: Scoping review

**DOI:** 10.1177/09697330251339417

**Published:** 2025-05-11

**Authors:** Ebin Arries-Kleyenstuber, Bernadette Dierckx de Casterlé, Kathryn Kynoch, Mary-Anne Ramis, Riitta Suhonen, Carla Ventura, Georgina Morley

**Affiliations:** University of Regina, Canada; 26657 KU Leuven, Belgium; Mater Health and JBI Queensland Center for Evidence-Based Health Innovation, Australia; Mater Health and JBI Queensland Center for Evidence-Based Health Innovation, Australia; University of Turku, Finland; University of São Paulo at Ribeirão Preto College of Nursing, Brazil; Center for Bioethics, Cleveland Clinic, USA

**Keywords:** COVID-19, ethics, moral distress, moral resilience, nurses

## Abstract

Nurses encountered a myriad of ethical challenges during the height of the COVID-19 pandemic, such as allocation of scarce resources, the need to balance duty of care with safety of self as well as visitation restrictions. The impact of these challenges on the nursing workforce requires investigation. The aim of this review was to scope and describe the reported literature on ethical challenges faced by nurses during the COVID-19 pandemic, including contextual characteristics and strategies reported to address these challenges. The review was conducted in accordance with JBI methods for scoping reviews and reported using PRISMA-ScR guidance. A published protocol guided conduct of the review. The following databases were searched for eligible studies from November 2019 to January 2023: PubMed, CINAHL, Ovid, PsycINFO, the Cochrane Library, and Scopus. No language restrictions were applied. Studies were reviewed for inclusion by two independent reviewers, and a data extraction form was developed to extract data relevant to the review questions. Results were analyzed and presented according to the concepts of interest, using tables, figures, and supporting narrative synthesis. After searching the databases, 2150 citations were retrieved with 47 studies included in the review. Studies represented 23 countries across five continents. Most of the studies used qualitative designs. Ethical challenges were described in several ways, often without appealing to common ethics language or terms. Few studies reported on strategies to address the specific challenges, which may reflect the dynamic nature of the pandemic. The scoping review highlights the complex and, at times, overwhelming impact of ethical challenges faced by nurses across the globe during the COVID-19 pandemic. Findings from the review can be used as a basis for further research to explore, develop, and implement strategies to address ethical challenges faced by nurses during future public health crises.

## Introduction

The COVID-19 pandemic impacted the nursing workforce globally. The aftermath of the pandemic has been reported to have affected the physical, emotional, and psychological health and well-being of nurses who were required to care for patients in challenging conditions with limited resources.^1–3^ Negative impacts to staff were reported from across various care settings such as aged care,^4,5^ home care,^6^ and hospice settings,^7^ as well as within acute hospital environments including, but not limited to, intensive care units,^8^ emergency departments, and critical care settings.^9^ The many challenges that arose during the pandemic, such as the increased physical and psychosocial burden of care, facing unmet needs, receiving insufficient support, having concerns about the workplace environment, and struggling with fear and accepting uncertainty,^10^ collectively demonstrate some of the multi-dimensional challenges that nurses faced during this time.

Growing evidence has also identified the negative impact on nurse’s moral well-being, with links to increased levels of moral distress, depression, and anxiety.^11,12^ Prior to the pandemic, Haahr et al.^13^ reported on organizational challenges that nurses faced in their daily practice, which impeded providing ethical and holistic care, such as workload imbalances and hierarchical structures. During the waves of the pandemic, these challenges were heightened and at times, insurmountable. Many more studies have elucidated challenges faced by nurses when trying to fulfill their ethical care responsibilities during the pandemic. A systematic review by Fernandez et al.,^14^ included 13 studies reporting on the experiences of nurses working in acute care settings during the pandemic and highlighted the high levels of stress and anxiety. Nurses were reported to experience a strong sense of duty to patients but needed to balance their obligation to patients with the safety of their own families.^14^ A review by Oh and Gastmans^3^ synthesized data from 26 qualitative studies, finding two main themes. Firstly, the authors reported on the “ambivalent emotions” and tensions experienced by nurses when caring for patients with COVID-19. They described how nurses drew upon their “moral imagination” to express compassion towards patients but at the same time experienced uncertainty in relation to the unfolding public health crisis.^3^ Secondly, the review highlighted many ethical issues faced by nurses, which impacted their ability to care for themselves and their patients with dignity and respect.^3^ The review further elucidated organizational and societal barriers that nurses were required to overcome, along with physical, mental, and emotional challenges that impacted ethical obligations.

Due to the overwhelming amount of research conducted during the pandemic that described nurses’ experiences, we identified a need to capture the ethical challenges that arose. We sought to scope the range and source of information and adopted a broad and inclusive understanding of the concept of “ethical challenges.” While the studies referenced above reported on experiences and ethical challenges nurses encountered during the pandemic, no published scoping review has also identified the strategies implemented by nurses or health services, to address the ethical challenges. Therefore, the objective of this scoping review was to scope and describe the existing empirical evidence on ethical challenges faced by nurses while caring for patients during the COVID-19 pandemic, along with any reported strategy implemented to address those challenges. Understanding these concepts can facilitate the selection and implementation of preventive, supportive, and remedial strategies to proactively address and deal with ethical challenges in future pandemics.

## Aims

This scoping review aimed to describe the literature that captures the nature and scope of ethical challenges faced by nurses during the COVID-19 pandemic, with a focus on the contextual characteristics of ethical challenges and strategies reported to address these challenges. The following research questions guided the scoping review methods, data synthesis, and results:

(1) What ethical challenges were reported as being faced by nurses while caring for patients during the COVID-19 pandemic?
(2) What were the contextual characteristics of the ethical challenges faced by nurses while caring for patients during the COVID-19 pandemic?
(3) What were the reported strategies used by nurses to address ethical challenges?

## Methods

The scoping review was guided by JBI methods^15^ and an *a priori* published protocol.^16^ The review is reported according to the Preferred Reporting Items for Systematic Reviews and Meta-Analyses extension for Scoping Reviews (PRISMA-ScR) guidance.^17^

### Population

The population of interest for this review was nurses from any clinical setting or geographical location. For this review, “nurse” was defined as an individual who, regardless of the level of education or license/registration status, is:

…prepared and authorized to (1) engage in the general scope of nursing practice, including the promotion of health, prevention of illness, and care of physically ill, mentally ill, and disabled people of all ages and in all health care and other community settings… and (3) participate fully as a member of the health care team.^18^

As the aim of the review was to include studies reporting on ethical challenges experienced by nurses, studies where data could not be separated by profession were excluded.

### Concept of interest

The concept of interest for this review was the ethical challenges faced by nurses during the COVID-19 pandemic. Ethical challenges were defined as ethical conflicts, ethical dilemmas, ethical issues, moral challenges, moral uncertainty, or difficult choices.^19^

### Context

Studies that reported on nurses working within any clinical care setting, such as acute hospitals, nursing homes, residential aged care facilities, or specific units, such as intensive care, cancer care, or specific COVID-19 wards, were considered for inclusion.

### Types of sources

We considered quantitative, qualitative, and mixed-methods study designs. Due to the rapid nature of reporting during the pandemic, we included a range of research synthesis publications, including integrative and systematic reviews. For qualitative studies, only studies with themes that had specific verbatim quotations from nurses were included.

### Search strategy

A three-step search strategy was undertaken to locate both published and unpublished literature. An initial limited search of PubMed and CINAHL (EBSCO) was conducted, followed by an expanded search using all identified keywords and index terms used to describe the articles. The text words contained in the titles and abstracts of relevant articles, and the index terms used to describe the articles were then used to develop a comprehensive search strategy that was adapted for each included database and information source.

The databases searched included PubMed, CINAHL (EBSCO), Ovid, PsycINFO (EBSCO), the Cochrane Library, and Scopus. Sources of unpublished studies and grey literature included ProQuest Theses and Dissertations (Ovid), the Canadian Agency for Drugs and Technologies in Health (CADTH), and Grey Matters. Articles published from November 2019 to January 2023 were included to align with the publication of literature specific to the COVID-19 pandemic. Papers published in any language were eligible for inclusion in the review. Papers not published in English were either assigned to a member of the research team with fluency in the language or translated using online translation software. As a review team, we agreed to use the translation software Google Translate as it was publicly accessible. Google Translate uses neural machine translation, employing artificial neural networks and deep learning techniques to translate sentences, improving fluency and accuracy across various languages. Citations of four sources of evidence in German, Italian, Arabic, and Korean languages were identified. Upon retrieval of the full text, it was uploaded and run through Google Translate. A team member with fluency/ proficiency in the language of translation had to confirm the accuracy of the translation, ensuring the translation reflected the original meaning and intent. The Korean language paper was excluded for focusing on the wrong concept following two independent reviewers’ review of its title and abstract. The Italian language paper was excluded for focusing on the wrong participants and wrong concept. No full text for the Arabic language paper was accessible or retrievable, and its abstract provided very little information to be deemed useful for the objectives of this review.

### Study selection

Following the search, all identified records were collated and uploaded into EndNote v.X19 (Clarivate Analytics, PA, USA) and Covidence, and duplicates were removed. An initial selection of 25 studies was pilot tested by two reviewers to ensure congruence with inclusion criteria and to enable discussion and/or any modifications to definitions.^15^ Titles and abstracts were then screened in Covidence by two independent reviewers for assessment against the inclusion criteria for the review. Papers that were deemed potentially relevant were retrieved in full and their citation details imported into a Microsoft Excel file (Redmond, Washington, USA). The full text of selected citations was then assessed in detail against the inclusion criteria by two independent reviewers. At this stage, reasons for exclusion of full-text papers that did not meet the inclusion criteria were recorded.

To ensure consistency in decision-making regarding sample selection, we developed our inclusion criteria based on our review questions and objectives, using the JBI recommended PCCS elements—Population/ Participant, Concept of Interest, Context, and Type of Sources. Therefore, the parameters of our inclusion criteria are the right population/participant, right concept, right context, and right type of source/design. Detailed descriptive information on the eligibility criteria (inclusion and exclusion criteria) for sample selection is listed in Table 1.

**Table 1.** Eligibility criteria: inclusion criteria and exclusion criteria.

Inclusion criteria	Exclusion criteria
Right Population/Participant:	Wrong Population/Participant:
• Nurses	• Not nurses
oNurse Practitioners (NPs)	• Other healthcare professionals
oClinical Nurse Specialists	• Student nurses
oRegistered Nurses (RNs)	
oLicense Practical Nurses/Enrolled Practical Nurses (LPNs/EPNs))	
oNursing Assistants/Enrolled Nursing Assistants (NAs/ENAs)	
Right Concept:	Wrong Concept
• Ethical challenges	• Not ethical challenges, ethical conflicts, ethical dilemmas, ethical issues, moral challenges, moral uncertainty, or difficult choices
• Ethical conflicts	
• Ethical dilemmas	
• Ethical issues	
• Moral challenges	
• Moral uncertainty	
• Difficult choices	
Right Context	Wrong Context
• Any clinical setting (healthcare setting) nurses work/care for patients	• Non-clinical settings
• Acute hospitals, nursing homes, residential aged care facilities, or specific units, such as intensive care, cancer care, or specific COVID-19 wards (primary health care or community)	• Educational institutional settings
	• Science laboratories
Right Source/Types of Design	Wrong Source/Type of Design
• Primary research studies employed empirical design:	• Types of sources of information not involving empirical research
oQualitative design	oDiscussion papers
oQuantitative design	oEditorials
oMixed-method study design	
• Research synthesis publications:	oOpinion papers
oIntegrative reviews	
oSystematic reviews	
• Published between November 2019 and January 2023	
• Unpublished studies:	
oTheses and dissertations	

In our scoping review, we took a broad view of definitions (i.e., nurse and nursing) to be inclusive rather than exclusive, especially considering that the COVID-19 pandemic was a globally shared stressor impacting all nurses regardless of status. However, student nurses were excluded because we took the view that this was a unique population, and, in many countries, they were withdrawn from clinical practice due to safety concerns. Right population/participants included nurses prepared at all levels of education authorized to care for patients during the COVID-19 pandemic, which included nursing assistants who are regulated by the same licensing bodies as nurses in many countries. Studies where data could not be reported by profession/occupation were excluded since the nurse-specific data could not be extracted. The right concept included studies focusing on the broad and inclusive concept of interest “ethical challenges,” which comprised ethical conflicts, ethical dilemmas, ethical issues, moral challenges, moral uncertainty, or difficult choices. Studies that did not specifically focus on these key concepts were excluded. The right context included any clinical setting (healthcare setting) where nurses work such as acute hospitals, nursing homes, residential aged care facilities, community settings, or specific units, such as intensive care, cancer care, or specific COVID-19 wards. The right type of source/design included primary research studies that employed an empirical design, including qualitative, quantitative, or mixed-method study design. Due to the rapid nature of reporting, we also considered a range of research synthesis publications, including integrative and systematic reviews. Other sources of evidence included unpublished empirical research dissertations. Two dissertations were retrieved but eventually excluded because they focused on the wrong concept. Other sources of information (i.e., discussion papers, editorials, and opinion papers) that did not involve empirical research on the concept/phenomenon of interest were excluded.

### Data extraction

Included papers were divided between the authors, and data was extracted from papers included in the scoping review by two independent reviewers using a data extraction tool developed by the reviewers. The data extracted included specific details about the research design, nurse participants, their practice context and setting, geographical location, and content relevant to the review questions. The draft data extraction tool was piloted and revised as necessary by the reviewers while extracting data from each included paper. Any disagreements between reviewers during the selection process or data extraction were resolved through discussion or with a third reviewer.

### Data analysis and presentation

Data from the included studies is presented in a narrative summary of identified overarching categories and subcategories, after examining the concepts of interest. Tables, figures, and graphs have been utilized to provide a visual display of the results in line with the objectives and questions of the scoping review.^20^

Following the JBI scoping review framework,^20^ our analysis and presentation were meticulously guided. Two reviewers independently conducted the analysis, resolving any disagreements through discussions with a third reviewer or the review team. We moved through the analysis process iteratively. Initially, two authors categorized the data and discussed the results with the review team. Using this coding framework, we organized the data and, through iterative analysis, identified relevant additional categories and subcategories, which were organized into overarching categories. Scoping reviews are descriptive and aim to map rather than reinterpret the available evidence; therefore, we aimed to provide a descriptive analysis that aligns with this methodological approach.^20^ After examining the concepts of interest, data from the included studies is presented in a narrative summary of identified overarching categories and subcategories. Tables, figures, and graphs have been utilized to provide a visual display of the results in line with the objectives and questions of the scoping review.^20^

## Results

### Study inclusion

**Figure 1.**
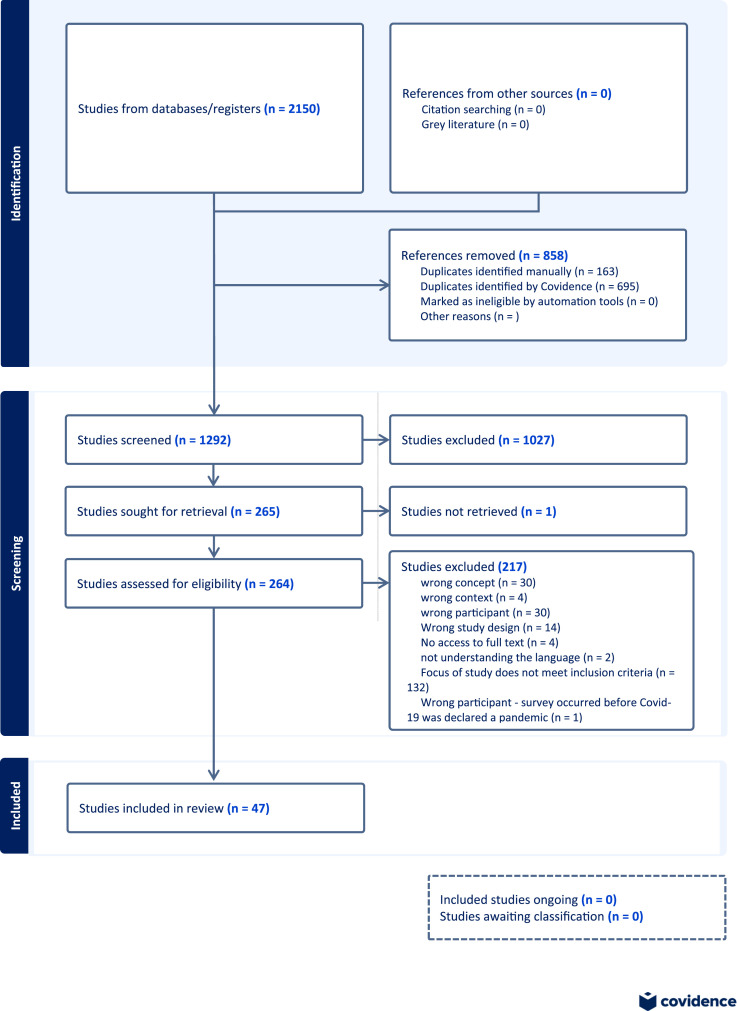
PRISMA flow chart of search and selection process.

After searching the databases, 2150 citations were found. After removing duplicates (*n* = 858) and screening for eligibility according to the inclusion criteria (*n* = 1292), the team analyzed 264 full-text articles. Finally, 47 articles judged against the full inclusion criteria were found suitable for inclusion in the review. Figure 1 outlines the search and selection process.

### Characteristics of included studies

Of the 47 included studies, 39 reported qualitative designs using various data collection methods, including focus groups, in-depth interviews, and free text fields within surveys. Three quantitative studies used cross-sectional methods, and five review papers were also included. The review papers reported a total of 94 articles, and there was some overlap across the reviews. It is important to note that some of the review papers included editorials and opinion pieces that reflected the types of articles reported during the pandemic, particularly in the early stages.

**Figure 2.**
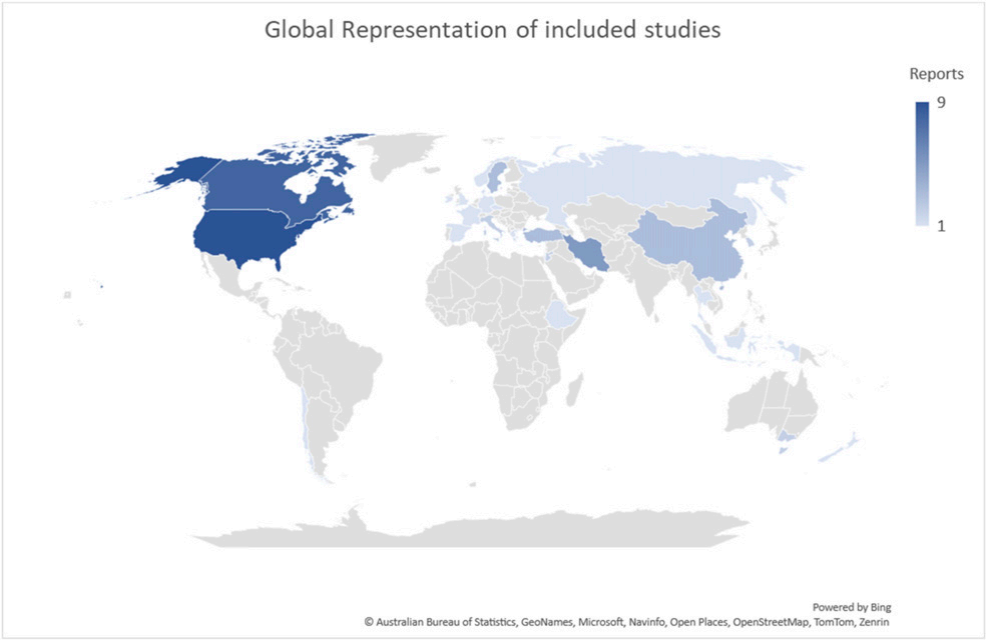
Global representation of included studies.

The 47 included studies represented a range of countries. Most included reports were from the United States of America and Canada. Figure 2 presents a global representation of included studies (*n =* 47).

The 47 studies included registered nurses, nurse managers, chief nursing officers, nursing assistants, community nurses, and midwives. Contexts of practice included intensive care units, community centers, acute hospital wards, and units, as well as COVID-specific units, emergency departments, palliative care units, and general medical/surgical wards.

## Narrative of findings

**Figure 3.**
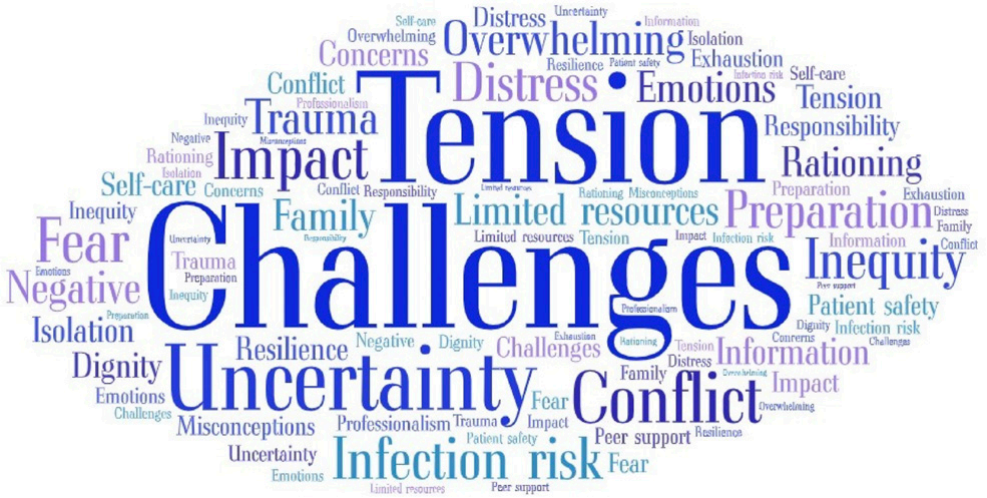
Word cloud representation of categories and sub-categories.

The word cloud (https://wordart.com/) presents a broad, visual summary of the most frequently reported phrases and text words within the category findings. To determine word frequencies, categories and subcategories were reviewed according to the number of studies within each section and associated repeated text words, with larger font indicating more frequent presentation (Figure 3). In the following narrative, further explanation is provided regarding the overarching categories and sub-categories according to the concepts of interest: ethical challenges, contextual characteristics, and reported strategies.

### Overarching category 1: conflict and tensions between primary commitments to patients with pandemic-related constraints

#### Category i: ethical tensions when balancing risk of infection

Twenty-two studies described several ethical tensions that arose as nurses tried to balance their responsibilities to patients with minimizing the risk of infection.^4–6,21–39^ Nurses described multiple times where they had to compromise the quality of care provided to patients to minimize infection risk. For example, patients died alone without loved one’s present^22–26^ and in care home settings, nurses described medicating or isolating patients to prevent them from interacting with other patients.^4–6,21,37^ Nurses described how some of the infection control measures created internal conflict^38^ and contributed to moral distress.^33^

#### Category ii: negative impact on patient care

Nurses cited various reasons for the negative impact on patient care that all stemmed from the challenging context and demands of the COVID-19 crisis. The negative impacts on patients ranged from an inability to provide care that upheld patients’ basic rights,^40,41^ that was patient-centered,^22,42^ family-centered,^37,43^ holistic,^31,43,44^ or relational.^37,45–47^ Nurses described challenges regarding communication and contact between patients and their families, which was then especially traumatic when patients died alone.^8,25,28,32,33,44,45^ Nurses provided a number of different reasons for poor quality care, such as insufficient information or knowledge about COVID-19,^21,27,30,45^ responsibility for non-nursing tasks leaving less time for nursing tasks,^48,49^ and arbitrary visitation restrictions.^22,24,26^ Notably, many nurses expressed feeling unprepared for the demands placed on them, which compromised their ability to provide safe, effective care.^4,28,33,35–38,41,43,47,49–51^ The combination of immense pressure and the inability to alleviate patients’ heightened fear and anxiety during the pandemic further intensified their sense of professional inadequacy^41^ and moral distress,^33^ which are described in overarching category 3.

#### Category iii: organizational and contextual challenges

The third category captured the contextual and organizational challenges that exacerbated the ethical challenges that nurses encountered when trying to balance their commitments to patients with infection control measures.^4,8,26,28–30,32,41,47,49,52^ Nurses described challenges related to frequent policy changes that arose due to the clinical uncertainties about COVID-19^8,29,30^ and the multiple disparities they experienced in relation to other healthcare workers.^4,26,30,32,41,52^ In one study,^26^ an experienced nurse manager described the negative impact that inequities between nurses and physicians had on nursing morale: “The expectation that the providers only went into the room one time per shift definitely impacted the nurses’ thoughts on the support that they had. They avoided it [going into rooms] at first. Like there aren’t as many providers as there are nurses. We have to keep everybody safe. Not only were my nurses doing that, they were taking out garbage. They were changing the sharps containers. Everything was being put on them because they were the ones providing the most care at the bedside” (p. 2409–2410). Similarly, Menard et al.^28^ described how one nurse had stated that she felt like a “sacrificial lamb.” The sense that nurses were isolated and left to fend for themselves and patients permeated this category.^4,26,28,30,32,32,41,47,47,52^

#### Category iv: resource shortages

Six publications captured the ways in which resource scarcity constrained nurses from their ability to fulfill their primary commitments to patients.^28,29,33,39,44,45^ Scarce resources included staffing shortages and a lack of Personal Protective Equipment (PPE), medical equipment, and facilities.^28,29,33,39,44,45^ The lack of PPE and instruction to re-use PPE was, in particular, concerning for nurses because this increased their risk of infection and contributed to unsafe working conditions, which were further exacerbated by nursing shortages, adding to feelings of anger, stress, and moral distress.^28,29,33,45^ Smith et al.^44^ captured four sub-categories of moral distress in their interviews with 88 women healthcare professionals and described how lack of PPE created a “double distress” because nurses and midwives did not feel safe at work and then had to risk infection of their loved ones at home.

#### Category v: increasing and overwhelming workloads

Eight publications provided data that indicated nurses felt unable to fulfill their responsibilities to patients and provide quality care because they were overwhelmed with their workloads.^21,28,30,33,38,39,45,48^ Nurses encountered increased workloads for many reasons. Nurses reported increased patient number and acuity,^39^ insufficient staffing,^30^ challenges with pivoting to team models of nursing,^33^ redeployment,^28^ and responsibility for non-nursing tasks such as housekeeping and blood draws.^28,48,49^ Some nurses stated that efforts to minimize infection risk meant that they took on these tasks since they were expected to cluster care,^28,48^ emphasizing another injustice between professionals.^26^ Nurses had to approach their work in a task-oriented way,^33^ which then impacted their relationships with patients.^47^ Kelley et al.^30^ reported that “nurses felt they were ‘becoming everything’ to patients and yet felt organisations viewed nurses as ‘expendable’” (p. 2172). Jackson et al.^45^ described overwhelming workloads as a risk factor for negative experiences and moral distress, which was supported by several authors (Table 2).^21,38,48^

**Table 2.** Ethical challenges captured in categories and sub-categories from included studies.

Overarching category	Category and sub-category
1. Conflict and tensions between primary commitments to patients with pandemic-related constraints	i. Ethical Tensions When Balancing Risk of Infection
	oPatients medicated and/or isolated to prevent interaction with other patients^4–6,21,37^
	oPatient’s dying alone^22–26^
	oInsufficient access to PPE^23,27–32^
	oInfection control measures contributed to moral distress^33^
	oIt should not be mandatory to care for patients with COVID-19^34,35^
	oFear of infecting loved ones^36^
	oResponsibility to ensure patients adhere to infection control measures^37^
	oFeeling conflicted about prioritizing self and responsibility to patients^38^
	oChallenges providing good care in response to sudden changes in practice^37^
	oFamily visits with the patients^39^
	oConflict with families regarding appropriate use of resources^33^
	ii. Negative Impact on Patient Care
	oInsufficient information or knowledge to provide adequate care^21,30,45,49^
	oPatient’s rights were violated^40,41^
	oVisitation restrictions^37,58^
	oArbitrary visitation restrictions^22,24,26^
	oProvision of non-beneficial treatments “futile care”^29^
	oPPE created barriers between nurse and patient^37,45–47^
	oNot enough time to care properly^48,58^
	oInsufficient quality of care^26,38,47^
	oRequired to take on additional non-nursing tasks^48,49^
	o “Rituals of death” including visitation and provision of spiritual care could not be provided^25^
	oUnable to provide “holistic care”^31,43,44^
	oUnable to provide patient-centered care^22,42^
	oUnable to provide family-centered care^37,43^
	oRapid changes to patient care^28^
	oSurrogate decision-making^26^
	oChallenges providing good care in response to sudden changes in practice^37^
	oInability to provide care to other patients, besides those with COVID-19^33,49^
	iii. Organizational and Contextual Challenges
	oFrequent policy changes^8,29,30,44^
	oDisparities between how nurses and other healthcare workers were treated^26,30,32,41,52^
	oBeing left alone with responsibilities^4^
	oStaffing shortages^57^
	oRequirement to complete additional non-nursing work^49^
	oLack of management response and support^28,32,47^
	oChallenges with maintaining quality care when protocols changing rapidly^44,47^
	oChallenges associated with changes in the work environment^28,30^
	oLack of support from the healthcare organization^30,47^
	oFrustration and concern about poor communication^30^
	oImplementation of team nursing care delivery model^33^
	iv. Resource Shortages
	oPracticing in the midst of scarce resources, which included staffing shortages and inadequate access to PPE^29,33,39^
	oAnger and stress associated with resource scarcity and the need to re-use/ conserve PPE^28,33,45^
	oLack of resources and staffing shortages increased the risk of contagion^29^
	oReusing PEE created unsafe working conditions and negatively impacted patient care^28,44,45^
	oLack of PPE for midwives, resulted in increased risks for families^44^
	oICUs were frequently short staff, which left nurses feeling exhausted^29^
	v. Increasing and Overwhelming Workloads
	oIncreased workload^21,30,33,45^
	oTask-oriented model of care^33^
	oIncrease number of patients^39^
	oInadequate staffing creating more work^30^
	oAdditional responsibilities for nurses (e.g., housekeeping and blood draws) as changes led to staffing restrictions^28,48^
	oExperienced that they lack prerequisites to manage the extreme workload^38^
	oBeing redeployed to high-demand areas to manage the first surge of the pandemic without adequate preparation and training^28^
2. Challenges to nurses competence	i. Lack of Information, Knowledge, and Preparation
	oDid not feel professionally competent^4,35,36,41,49^
	oInsufficient information or knowledge to provide adequate care^21,30,45,49^
	oLack of preparation and training^28,30,45,49^
	oLack of information contributed to moral distress^30,33,38,41,44^
	oLack of information contributed to worry and stress^41^
	oClinical dilemmas due to lack of correct information about COVID-19^27,36,50^
	oRedeployment to unfamiliar areas^28,33,45,47,51^
	oChallenges providing good care in response to sudden changes in practice^37^
	oExtreme workloads challenged nurses’ decisions^4,38^
	oInsufficient responses to urgency requirements of the situation^41^
	oChallenges with maintaining quality care when protocols changing rapidly^29,30,37,44^
	oBeing overwhelmed by the breadth of the COVID-19 illness^33^
	oSafely donning and doffing PPE was time-consuming, leaving less time for interacting with patients at the bedside and managing basic care^28^
	oNewly graduate nurses feeling lost and inexperienced on entering the emergency department environment during a pandemic^45^
	ii. Uncertainty when Faced with a Novel Disease
	oDon’t’ know what is ethical and what’s not^30,38^
	oLack of confidence in knowing the right approach to an ethical issue or even how to address a particular ethical situation, often leading to substantial confusion^30^
	oLack of knowledge and uncertainty regarding how to treat a new illness^30,33,41,43,49^
	oDealing with uncertainty^21^
	oUncertainty about what to do due to frequent policy and guideline changes^28,29^
	oSome nurses did not know what, whom, and how to prioritize^38^
	oFacing the unknown disease^50^
	oRapid onset of chaos and confusion^28^
	oLack of communication^21,30,33,44^
	oMoral uncertainty distress about how to best protect patients due to constantly changing information regarding COVID-19^44^
	oInstitutional and policies constraints leading to moral distress^29,33,48^
3. Moral distress and emotional distress related to ethical challenges	i. Causes of Moral Distress
	oFear for own safety and patient safety^36,48^
	oFear for own safety and their loved ones^22,44^
	oProvision of non-beneficial treatments^29,33,36,53^
	oInsufficient time to provide quality care^22,33,48,51,52^
	oPatients dying alone^29,30,33,51^
	oPatients dying without dignity^53^
	oPatients left intubated when withdrawing life-sustaining treatments instead of extubating^30^
	oFeeling unsafe due to risk of infection and lack of PPE^23,33^
	oMagnitude of deaths^30^
	oLack of knowledge and uncertainty about treating a new disease^33^
	oFear of exposure to the virus leading to suboptimal care^33^
	oTeam model of nursing care that caused intra-professional tensions and miscommunications^33^
	oInfection control policies negatively impacting patient care^33^
	oPracticing within crisis standards of care^33^
	oResource scarcity^33^
	oHard decisions^52^
	oDisparities between how nurses and other healthcare workers were treated^52^
	oInsufficient staffing^51,52^
	oVisitation restrictions^22,48,51^
	oInequities with visitation restrictions^22^
	oConflict with family members and other healthcare professionals^48^
	oSurrogate decision-making^26^
	oWatching patients suffer^51^
	oMoral constraint distress at work and home^44^
	oWork: negative impact on quality of patient care and lack of PPE constraining ability to keep patients safe
	oHome: lack of PPE constraining ability to keep their family safe, inability to support their child’s education, and unpaid care responsibilities
	oMoral conflict distress at work and home^44^
	oWork: conflict with manager who worked from home and did not understand the realities
	oHome: lack of flexibility at work
	oMoral dilemma distress at work and home^44^
	oWork: challenges navigating provision of standard of care and maintaining COVID-19 protocols
	oHome: balancing occupational risk and responsibility for dependents’ well-being
	oMoral uncertainty distress at work^44^
	oWork: constantly changing information regarding COVID-19 made it difficult to know how to best protect patients
	ii. Effects of moral distress
	oIntention to leave^52^
	oEmotional effects^6,48,52^
	oSadness, stress, and anger^48^
	oFeeling ashamed, guilty, and helpless^6^
	oPhysical effects^52^
	iii. Emotional and Ethical Challenges
	oSocial isolation^21,36,48^
	oWhether to allow visitation at end-of-life^21^
	oConfronted with ethical and moral challenges^38^
	oMoral uncertainty^30,44^
	oMoral dilemmas^27,30,31,44,46^
	oNurse participant reported feeling alone in trying to resolve dilemmas, which contributed to many negative feelings and emotional responses^30^
	oNurses faced ethical dilemmas related to end-of-life of corona virus patients^21^
	oEthical dilemmas reflect values such as professional autonomy, beneficence, non-maleficence, equality, and justice^27^
	oNurse participants were unable to articulate the ethical principles or values that conflicted^30^
	oMoral breakdown^55^
	oMoral identity disruption^56^
	oEmotional and ethical challenges^21^
	oEmotional burden, communication difficulties and restrictions, dealing with uncertainty, and working when the medical knowledge is only starting to develop^21^
	oConcerns with beneficence-nonmaleficence, autonomy, and justice^27,31^
	oDealing with family caregivers who complained about shortened visiting hours and strict visiting limitations^46^
	oPatients and families could not see each other and experience a dignified death^46^
	iv. Emotional Distress
	oSpirituality, feelings, and emotions^42^
	oUniqueness of the lived experience, indescribable, and warlike experience^42^
	oPsychological casualties: fear, stress, burnout, and isolation^8^
	oBurnout^6,51^
	oSeeing patients suffer^45^
	oNurses feeling abandoned with decision-making^4^
	oInsufficient staffing^4^
	oProviding futile care^29^
	oWitness to families’ grief^29^
	oPatient’s dying alone^29^
	oNegative feelings and emotions^30,57^
	oRisks associated with insufficient safety precautions resulted in nurses feeling disrespected by their healthcare organization^30^
	oPride, isolation, and fear^36,42^
	oPositive emotions^30,36,42,57^
	v. Moral Injury
	oBetrayal/community, isolation, and alienation^32^
	oMoral injury^30^
4. Inability to meet responsibilities: ethical, professional, and decision-making	i. Challenges Meeting Responsibilities
	oInability to provide “proper care”^48^
	oWitnessing inadequate provision of care^33^
	oCare of patients (palliative care) perceived as inappropriate^53^
	oNurses witnessed other colleagues lessen the frequency of performing nursing tasks due to fear of being infected^45^
	oResearch on patients and the risk of disclosing patients’ identities^39^
	ii. Challenges to Decision-Making
	oLimited resource forced allocation decisions^28,39^
	oImpact on decision-making^4^
	oChallenging and unbearable decisions, some perceived as dishonest^4^
	oNurses reported being left out of important decision-making^4,31^
	oNurses felt left out of decisions regarding pandemic policies that had implications for their practice and for patient care^24^
	oNurses wanted more input into decisions regarding patient care and unit policies^29^
	oNurses’ voices not “heard” in care plan decisions^33^
	oChallenges related to making treatment decisions^39^
	oBalance between the right to choose and beneficence in the implementation of infection control measures in care homes^5^
	oDisagreements between nurses and physicians regarding appropriate use of treatments, including what they considered “futile”^33^
	iii. Professional Autonomy and Professional Ethics
	oThey felt powerless and had no autonomy to make decisions^52^
	oFeeling of de-professionalization because they are unable to provide professional care^4,48^
	oNurses cannot fulfil their moral and ethical values^48^
	oNurses being left alone and overwhelmed by various responsibilities^4^
	oLimited autonomy in choices over infection control measures^5^
	oLack of autonomy and feelings of powerlessness^29^
	oProfessional impact—positive impact related to nurses feeling well-prepared, professionally accomplished, and appreciated by the public. Negative impact related to feeling fearful and under-prepared, especially newly graduated nurses^45^
	oThreats to professional values^43^ a. The risk of declining quality of care^43^
	b. Stigmatized public image (“as infected”)^43^
	oConflict with other professionals^33,48^
	oInterprofessional collaboration is experienced as stressful, especially when nursing expertise is questioned^48^
	oProfessional ethics^41,45^
	oInsufficient responses to urgency requirements of the situation^41^
	oLow sense of responsibility in nursing services^41,45^
	oFailing in fulfilling ethical obligations^41,45^
	oLow sense of responsibility to patient outcomes^45^
5. Human (patient) rights, social justice, and equity concerns	i. Neglected Patients and Family Rights
	oPatients unable to make decisions about treatment plans and were not always provided access to PPE creating personal safety issues^41^
	oNurses stated increasing feelings of neglected patient rights^45^
	oNurses felt forced to lie to patients^4,41^
	oResearch on patients and the risk of disclosing patients’ identities^39^
	ii. Loss of Dignity
	oLoss of patient dignity
	oDue to invasive procedures^36^
	oLack of privacy and inadequate end-of-life care in particular due to visitation restrictions and lack of equipment for post-mortem care^38^
	oLoss of patient dignity at end-of-life^28,53^
	oEthical challenges around respecting the dignity of the patient and their behavior^39^
	oInability to provide adequate quality of care^38^
	oLoved ones demonstrated dignity when grieving^36^
	iii. Inequality: Disparities between Professionals
	oDisparities between professionals
	oUnequal exposure to the infectious environment^26,41,52^
	oRole ambiguity between doctors and nurses resulting in nurses taking on additional work^41^
	oChallenges regarding the distribution of skilled and unskilled personnel^4^
	oUnfair distribution of PPE among units and healthcare workers^28,44^
	oBeing left alone with the responsibility^4^
	iv. Rationing Resources: Care and Resource Allocation
	oAllocation of scarce resources^23,28,33,39^
	oNurses and other medical staff had to decide who would benefit most from ICU beds, ventilators, extracorporeal membrane oxygenation (ECMO), cardiopulmonary resuscitation, and intubation^28^
	oRationing nursing care such as personal care and communication^48^
	oDisparities between patients with access to healthcare resources^30^
6. Concerns related to patient safety and nurse safety	i. Concern for Patients’ Safety
	oSafety concerns and increased risk of infection for patients and families^30^
	oConstant fear for patient safety^39,48^
	oUnable to ensure patient safety due to lack of access to PPE^44^
	oLack of experience and clinical skills for nurses to provide safe patient care^43^
	oUnclear legitimacy regarding infection control measures^5^
	oInevitable harms to residents’ psychological well-being^5^
	ii. Concern for Nurses’ Safety
	oFear for our own (nurses) safety^23,27,29,33,39,48^
	oConcerns for own and their colleagues’ safety^29,30^
	oConcerns exacerbated by lack of resources
	oShortage of PPE and rationing of equipment put nurses at further risk of infection^29^
	oLack of resources and staffing shortages increases their risk of contagion^29^
	oReuse of PPE created moral concerns about safety^28,45^
	oSafety concerns contributed to moral distress^23,36^
	oWorking under circumstances that put them at risk to their overall health and well-being^23^
	oPhysical symptoms from a pandemic environment^45^
	oPhysical discomfort, physical exhaustion from long PPE use, and lack of necessities such as food, fluid, and toilet breaks^45^
	oLack of full protection for nurses across health industry raises ethical questions on the extent of their duty, lack of personal protective equipment, and risk of failure of personal protective equipment^23^
7. Challenges and concerns regarding relational impact of the pandemic	i. Impact on Relationships at Home
	oGuilt as unable to spend time with and educate children at home^44^
	oSocial isolation^21^
	oBeing urged by family members to stay home^21^
	oAvoiding family due to fear^42^
	oWorkplace colleagues and patients felt more like family at times due to the intensity and closeness from working together^42^
	ii. Challenges and Changes in the Quality of the Nurse–Patient Relationship
	oChanging nature of nurses’ relationship with patients and families^39^
	oNurse–client relationship^23^
	oEthical challenges of the triangular nurse–client–family relationship^23^
	oMixed feelings^42^
	oIn some cases, very significant relationships were developed with patients due to criticality of patient conditions and time spent together^42^
	oNurses acted as relatives^42^
	iii. Communication Challenges and Disagreements between Healthcare Professionals
	oAdopting a team model of nursing care caused intra-professional tensions and miscommunication^33^
	oInadequate communication led to safety concerns^33^
	oChanges to care models often made without nurse involvement^37^
	oConflict with medical staff who did not want to enter care home^48^
	oFeelings of frustration and concern because of poor communication about equipment and staff shortages^30^
	oNurses felt disrespected by medical staff and expendable to the organization^30^
	oOne of the biggest challenges was communication between healthcare professionals^42^
	iv. Communication Challenges with Family Members that Negatively Impacted Relationships
	oNursing dynamics within revolving teams^33^
	oFeeling uncomfortable outside the work environment^42^
	oCommunication difficulties and restrictions^21^
	oInadequate communication^33^
	oLack of communications regarding care plans^33^
	oNurses reported feeling frustrated and concerned about poor communication^30^
	oWhen the outbreak first happened, there was no communication about affected areas^44^
	oNurses conveyed their struggles in delivering bad news to families, informing them that their loved one may die, or had already died^45^
	oNurses stated acutely unwell patients were unable to communicate care needs or preferences, increasing feelings of neglected patient rights^45^
	oCommunication between relatives and patients took place exclusively at a distance, making the communication process difficult^36^
	oChanging nature of nurses’ relationship with patients and families^39^
	v. Strained Relationships with Management due to Lack of Transparency and Conflicting Communication
	oNurses’ highlighted communication between management and nurses, appear to be top-down^29^
	oIn light of limited evidence for decisions, clear communication was essential but was lacking^30^
	oLack of transparency regarding communication of decisions contributed to feelings of distrust^30^
	vi. Isolation and Lack of Emotional Support
	oDying in isolation is an extremely inhuman experience that COVID patients and their loved ones had to go through^22^
	oCOVID patient and family/friends have strong desire to see each other and get together^22^
	oBurying your loved one unclothed without saying goodbye is and intensively traumatic and stressful event^22^
	oNurses spoke of patient’s loneliness^37^
	oPlacing new resident into a sparsely furnished room in which they were forced to sit alone^4^
	oLack of emotional support^41^
	oThe horror to watch patients in isolation without emotional and physical support of family^37^
	oHospitalized critical COVID-19 patients are going through horror experience^22^
	oSome patients lost their family members resulting in lack of family relationships and negative attitude toward treatment^41^
	oTo decrease prevalence of infection, nurses and patients needed to maintain a certain distance when they communicate with each other, giving patients a sense of lack of security^41^
	oNurses felt social isolation from family, neighbors, and those in society^21,36^
	oLack of positive social relationships is a factor that contributed to development of moral distress in ICU nurses^36^

### Overarching category 2: challenges to nurses competence

#### Category i: lack of information, knowledge, and preparation

The COVID-19 pandemic highlighted significant ethical challenges related to nurses’ competence and confidence. Many nurses described feeling inadequately prepared for the demands placed on them, compromising their ability to deliver safe and effective care.^4,28,30,33,35–38,41,43,47,49–51^ Nurses described feeling overwhelmed by their responsibilities and unable to practice safely due to a lack of access to information about COVID-19, which created clinical dilemmas.^27,36,50^ Nurses described encountering barriers to providing safe and effective care because of redeployment to unfamiliar clinical areas^4,28,47,50,51^ working with nurses they did not know, or who lacked the appropriate qualifications or experience for specific areas, such as intensive care and emergency departments.^4,36,45^ The scarcity of information and training, along with misconceptions about the disease, exacerbated these issues, leading to confusion and limiting nurses’ abilities to make sound clinical and ethical decisions.^33,50^ The extreme workload and high-pressure environment further amplified feelings of inadequacy^4,35,36,41,49^ and moral distress.^30,33,38,44^

#### Category ii: uncertainty when faced with a novel disease

Nurses encountered many challenges to their competency due to the uncertainty generated by the limited and ever-changing information about how to prevent and treat COVID-19.^33,36,41,43,49,50^ Frequent changes in policy and guidelines,^28–30,44^ along with the lack of evidence-based best practices and treatment options, left many nurses unsure about how to deliver patient care.^30^ Inadequate communication further compounded the problem.^21,30,33,44^ Mènard et al.^28^ captured the rapid onset of confusion that characterized the initial months of the pandemic for nurses due to significant and rapid changes in structure and patient care. Some authors identified this uncertainty as moral uncertainty and provided data to evidence nurses’ experiences of moral-uncertainty distress,^30,33,38,44^ whereas other authors captured constraints as fear^36,48^ or external (institutional constraints and systemic policies) that contributed to moral-constraint distress.^29,33,44,48^

### Overarching category 3: moral distress and emotional distress related to ethical challenges

#### Category i: causes of moral distress

Authors captured various reported causes of nurse moral distress such as the provision of perceived “futile” treatments,^29,33,36,53^ the inability to provide sufficient quality of care,^22,33,48,51,52^ feeling unsafe for oneself and one’s family due to risk of infection,^22,36,44,48^ and lack of PPE.^23,33^ Some of the causes of moral distress captured were related to specific COVID-19 practices, such as patients being left intubated when withdrawing life-sustaining treatments at the end of life,^30^ visitation inequities,^22^ and the need to practice within crisis standards of care.^33^ Drawing on Morley et al.,^54^ Smith et al.^44^ interviewed women nurses and midwives and identified sub-categories of moral distress at home and at work. At work, moral-constraint distress occurred due to a lack of adequate staffing and inability to ensure parent and infant safety due to lack of PPE; moral-conflict distress occurred because of disconnection between those working with patients and managers working from home; moral-uncertainty distress occurred due to frequently changing information which made it difficult to know how to best protect patients; moral-dilemma distress occurred due to their inability to both provide an ethical standard of care and maintain COVID-19 protocols.^44^ One participant described going into therapy to try and mitigate feelings of guilt that occurred when she donned PPE prior to going into a deteriorating patient’s room: “… I couldn’t get into the room on time… I have to properly protect myself with a mask and the gloves. But meanwhile, the patient is crashing. And I just can’t get there on time. So, she ended up dying. And so, I’ve gone into counselling, so I can make sure I don’t feel the guilt” (p. 52).^44^ At home, moral-constraint distress occurred because nurses felt unable to meet their obligations to their own children due to lack of childcare availability and at-home schooling support; moral-conflict distress occurred when they engaged in conflict with employers because of lack of flexibility; and moral-dilemma distress occurred when then they had to make decisions about balancing their occupational risk at work and maintaining their families safety.^44^

#### Category ii: effects of moral distress

The explicit effects of moral distress were categorized as emotional,^6,48,52^ physical,^52^ and professional, specifically intention to leave one’s profession.^52^

#### Category iii: emotional and ethical challenges

Many authors did not use the specific term “moral distress” and instead described themes in their data as “emotional” and/ or “ethical challenges” that prompted an emotional response.^21,27,30,38,49,55,56^ In this category, there were multiple sub-categories identified as ethical challenges that triggered an emotional response from nurses, including social isolation,^21,36,48^ moral uncertainty,^30^ moral dilemmas,^27,30,31,44,46^ moral identity disruption,^56^ and moral breakdown.^55^ Other authors described concerns as explicitly related to specific ethical values. For example, Sperling^27^ captured nurses’ responses to various ethical dilemmas that reflect values such as professional autonomy, beneficence, non-maleficence, equality, and justice. Nurse participants were asked to report their level of agreement with ethical dilemmas such as “Nurses have a right to refuse to treat certain patients during the COVID-19 outbreak” and “every patient has a right to equal access to optimal treatment during the pandemic regardless of his/her age and health background” (p. 16).^27^ Sperling^27^ concluded that despite nurses facing great personal risk and emotional burden, the data gathered demonstrated a strong commitment to the provision of patient care, and they did not regret entering the nursing profession.

#### Category iv: emotional distress

Other authors described themes in their data as emotional challenges that were not specifically related to ethical challenges. De Benedictis et al.^42^ described nurses’ emotional experiences in two of their themes. The first theme, “Spirituality, Feelings and Emotions,” captured nurse participants’ experiences of “tiredness and fatigue,” “uncertainty,” “pessimism,” and “sadness.”^42^ The second theme, entitled “Uniqueness of the Lived Experience,” captured participants’ descriptions of how “unique” and “indescribable” their experiences were.^42^ Several studies described the sense of isolation that nurses experienced because they felt other individuals from outside of healthcare could not understand their daily experiences.^8,36,42^ In addition to negative feelings and experiences, authors also identified positive experiences.^30,36,42^ De Benedictis et al.^42^ described participants’ “mixed feelings” because, as with other nurse participants in other studies, they felt grateful because they had done all they could to contribute positively to the pandemic.^30,42,57^ Rosa et al.,^36^ Kelley et al.,^30^ and Chipps et al.^57^ also captured nurses’ sense of pride, in addition to hope and joy.^30^

#### Category v: moral injury

Only two studies specifically identified moral injury as experienced by nurses during the pandemic.^30,32^ Kelley et al.^30^ described moral injury as a prolonged or intensified type of moral distress and provided a verbatim quotation of a nurse participant describing a patient who died while still intubated as an instance where the nurse was forced to commit a moral wrong. In contrast, Song et al.^32^ captured moral injury as composed of isolation, alienation, and betrayal.

### Overarching category 4: inability to meet responsibilities: ethical, professional, and decision-making

#### Category i: challenges meeting responsibilities

Five studies reported nurses’ descriptions of challenges in meeting responsibilities to patients, including their inability to provide perceived appropriate or “proper” care^48,53^ and witnessing inadequate provision of care from others.^33^ Nurses described witnessing other colleagues lessen the frequency of performing nursing tasks due to fear of being infected^45^ and specific concerns about not maintaining confidentiality when conducting research.^39^

#### Category ii: challenges to decision-making

This category is composed of seven studies.^4,5,24,29,31,39^ Limited resources during the COVID-19 forced challenging allocation decisions.^28,39^ Hillestad et al.^4^ found that nurses described decision-making as “unbearable” and “dishonest” because they were told not to inform residents’ relatives when a patient was confirmed as having COVID-19. Nurses felt that such a lack of honesty would ruin the trust they had with relatives.^4^ Silverman et al.^33^ described disagreements that occurred between nurses and physicians regarding the appropriate use of treatments, including what treatments were considered “futile.” Many nurses reported being left out of important decision-making,^4,31^ stating that their voices were not “heard” in care plan decisions^33^ and especially regarding pandemic policies that had implications for their practice and patient care.^24^ Nurses described wanting to be involved in patient care and unit policy decisions.^29^

#### Category iii: professional autonomy and professional ethics

This category comprised eight articles in which nurses described various ways in which they felt either their professional autonomy^5,29,33,52^ or their professional identity or values^4,41,43,45,48^ were limited because of the pandemic. Rezaee et al.^43^ described how nurses experienced threats to their professional values due to declining quality of care and because they felt stigmatized by the public as “infected.” This finding was echoed by Jia et al.^41^ and Jackson et al.^45^ who described a diminished sense of professional ethics because nurses could not fulfill their obligations to patients. By contrast, Jackson et al.^45^ captured ways in which some nurses actually felt professionally fulfilled and appreciated by the public. This final category was also described by authors as one way in which nurses responded to ethical challenges and will be further described in overarching category 8.

### Overarching category 5: human (patient) rights, social justice, and equity concerns

#### Category i: neglected patients and family rights

This category is composed of five studies.^4,31,39,41,45^ Nurses identified ways in which patients’ rights were neglected during the pandemic, including the right to choose treatment plans, make decisions about their care, and access PPE.^41,45^ Both Jia et al.^41^ and Hillestad et al.^4^ described times when nurses felt forced to lie to patients or their family members for different reasons, violating their rights and therapeutic trust.

#### Category ii: loss of dignity

Authors captured dignity losses that patients experienced because of deviations from standard practices provided at the end-of-life.^28,36,38,39,53^ Mènard et al.^28^ reported that nurses felt that “The patients just didn’t receive that… support and dignity that they should receive [when they were dying] and that was kind of the worst of the worst experiences” (p. 46). Nurses described how patients were not able to say goodbye to their loved ones and they received inadequate post-mortem care due to infection control practices.^38^ By contrast, Rosa et al.^36^ described how nurses perceived patient’s loved ones as grieving with dignity because they could not be with patients at the end-of-life and instead said goodbye with a video call.

#### Category iii: inequality: disparities between professionals

Nurses described the various inequalities and disparities that existed between healthcare professionals in their roles. Nurses were expected to come into contact more regularly with patients with COVID-19, whereas physicians identified ways to minimize contact.^26,41,52^ A striking verbatim quotation reported by Atli Özbaş and Kovancı^52^ came from a Chief Nursing Officer who stated:

Physicians followed the events from a distance much farther during the process… because they did not enter the room of the patient unless they really had to …. Yet, my colleague has to enter the patient’s room and have one-on-one contact with the patient for the provision of care, who knows how many times a day. Their sadness became my sadness and my distress …. I agree with them but there is not much I can do (p.6).^52^

In addition to more frequent contact with patients with COVID-19, nurses also reported feeling isolated and abandoned with a lack of managerial support^4^ and unfair distribution of PPE among units and healthcare workers.^28,44^

#### Category iv: rationing resources: care and resource allocation

Several researchers identified rationing and allocation of health resources as a pressing social justice concern encountered by nurses during the pandemic.^23,28,33,39^ Disruption of supply chain equipment and personnel contributed to a lack of care and health resources, including personnel, PPE, medical equipment, and facilities.^23,33,39^ Without adequate resources, nurses and other medical staff had to decide who would benefit most from ICU beds, ventilators, extracorporeal membrane oxygenation, and cardiopulmonary resuscitation.^28^ In addition to rationing healthcare interventions, nurses reported having to ration nursing care activities such as providing personal care to patients and relational care such as communication.^48^ The need to implement allocation protocols, which in many circumstances were absent, contributed to nurses’ experiences of moral distress.^33,52^

### Overarching category 6: concerns related to patient and nurse safety

#### Category i: concerns for patients’ safety

Nurses described constantly fearing for patients’ safety for various reasons^5,30,39,43,44,48^ such as risk of infection,^30^ lack of access to PPE,^44,48^ concerns about the legitimacy of infection prevention measures such as social distancing,^5^ and an inability to provide adequate quality care because of inexperience and lack of clinical skills.^43^

#### Category ii: concern for nurses’ safety

Nurses described constantly fearing for their own personal safety^23,27,29,33,39,48^ and their colleagues’ safety.^29,30^ Nurse safety challenges were characterized by nurses as a pressing ethical challenge,^23,39^ which were attributed to unsafe working conditions created by the pandemic environment,^23,28,45^ including lack of PPE and reuse of PPE.^23,28,45,49^ Nurses raised ethical concerns about unsafe patient care^28,45^ and questioned the extent of their professional duties in the face of such challenging circumstances.^23^ Nurses described a lack of access to necessities for themselves, such as nutrition, hydration, and toilet breaks.^45^ The overall risks that nurses encountered to their overall health and well-being left nurses experiencing moral distress^23,36^ and questioning the extent to which their employer respected and valued them.^30^

### Overarching category 7: challenges and concerns regarding the relational impact of the pandemic

#### Category i: impact on relationships at home

Authors reported on tensions and changes in dynamics related to both their professional and personal relationships as a direct result of COVID-19 restrictions. Smith et al.^44^ reporting through a feminist lens, captured guilt experienced because of constraints that prevented women nurses from being able to care for their children and provide home schooling when needed. DeBenedictis et al.^42^ and Melnikov et al.^21^ found that nurses described feeling socially isolated from friends and family because of their potential exposure to the virus and some nurses were afraid to be near their family. DeBenedictis et al.^42^ also reported that workplace colleagues felt more like family at times due to the intensity and closeness of working together through such an overwhelming situation.

#### Category ii: challenges and changes in the quality of the nurse–patient relationship

Three studies reported on challenges and changes to nurses’ professional relationships with patients.^23,39,42^ DeBenedictis et al.^42^ reported on nurses having mixed feelings while caring for their patients. In some situations, the nurse became very close to their patient and took on the role of their loved ones, while in other situations, they minimized contact for fear of infecting their own family. Two other integrative reviews reported on the ethical challenges of maintaining the nurse–client–family relationship due to visitor restrictions, quarantining, and resource shortages.^23,39^

#### Category iii: communication challenges and disagreements between healthcare professionals

Five studies highlighted communication challenges and tensions between health professionals.^30,33,37,42,48^ Interprofessional conflicts emerged because of communication challenges, disagreements about patient care plans, including end-of-life decisions, and feelings of being disregarded by other team members.^21,30,33,42,48^ Begerow and Gaidys^48^ reported moral distress from relationship challenges and conflicts with residents and families within a geriatric care setting. These reported challenges impacted trust and confidence in professional relationships and in the nurse–patient relationship. However, alongside conflict and reported challenges, some authors highlighted the strengthening of professional relationships following sacrifices made to keep patients safe.^42^

#### Category iv: communication challenges with family members that negatively impacted relationships

This theme was raised in eight studies.^21,30,33,36,39,42,44,45^ One of the most pressing communication difficulties and restrictions^21^ was due to the dynamic nature of revolving nursing and healthcare teams,^33^ especially, a lack of communication regarding care plans.^33^ Rosa et al.^36^ reported that communication between relatives and patients took place exclusively at a distance, making the communication process difficult, especially in cases of poor compliance or an inability to communicate. Furthermore, adopting a team model of nursing care caused intra-professional tensions and miscommunications with families.^33^

#### Category v: strained relationships with management due to lack of transparency and conflicting communication

Two studies reported on the impact COVID-19 had on nurses’ relationships with their organization and managers.^29,30^ Lack of transparency in communication, rapidly changing direction, and decisions being made without nurses’ input were some of the reasons identified that led to mistrust in higher-level staff. Kelley et al.^30^ further highlighted that as limited evidence was available at the time for some organizational decisions, clear communication was imperative. Without this, safety was at times perceived by nursing staff to be compromised.

#### Category vi: isolation and lack of emotional support

Six studies described the various ways in which patients, their family members, and nurses experienced loneliness, isolation, and a general lack of emotional support during the pandemic.^4,21,22,36,41,47^ Voulstos et al.^22^ conducted a qualitative study focused entirely on nurse practitioners’ experiences of patients dying alone and reported the perception that it was extremely isolating and inhumane. Other authors captured the emotional impact of this on nurses who continued to care for patients who were struggling without family presence^4,22,41,47^ and in some circumstances, nurses tried to fill that emotional void.^42^ In addition to witnessing patients experience loneliness, nurses also reported feeling isolated from their own family and friends.^21,36^

### Overarching category 8: responses to ethical challenges

This overarching category answers the third objective of this scoping review study, which is to describe the reported strategies nurses used to address the ethical challenges they faced while caring for patients during the COVID-19 pandemic (see Table 3). Responses to ethical challenges are captured by five categories.

**Table 3.** Responses to specific ethical challenges and general pandemic responses.

Category 8	Sub-category
1. Morally effective care	oFollowed ethical codes to provide humanistic and empathic care^50^
	oRespected patients’ values and beliefs^26,50^
	oAdhered to their professional values by maintaining their conscience and commitment to the profession and society^50^
	oUse of professional discretion and benevolent compassion, empathy, and good will to mitigate ethical problems of dying alone^22^
	oSelf-discipline and prioritizing obligations to patients and profession^55^
	oOnly able to overcome ethical challenges by prioritizing their nursing duties above themselves^22,49,55,58^
	oRemote communication technology, taking measures to protect privacy and dignity in patients sharing rooms; providing holistic care; serving as mediators; allowing family members to bring small things for patients from outside; and “looking the other way” when family members visited loved ones^22^
2. Self-care strategies and resilience	oReading, watching TV and movies, and listening to podcasts^47^
	oWatching TV^57^
	oSpending time on hobbies and recreational activities^41,47^
	oObtaining support from family and friends^8,41,47,49^
	oVenting emotions, for example, crying^41,47^
	oAvoiding media about the pandemic/outbreak^45,47^
	oAvoiding overtime^45,47^
	oMaintaining routines^47^
	oSeeking help from a personal therapist^47^
	oEngaging in mindfulness, yoga, or prayer^8,45,47^
	oWriting^31,47^
	oAssuming a positive attitude^47^
	oExercising^8,28,31,45,47,57^
	oShowering^47^
	oResting and sleeping^47^
	oHaving a balanced diet^45,47^
	oTime with pets^28^
	oGardening^28^
	oBeing creative^57^
	oMindfulness^57^
	oMeditation and prayer^45^
	oResilience^57,66^
	oMoral resilience^26^
	oFlexibility and creativity^66^
3. Collegial and team peer support	oPeer support^26,28,33,57^
	oIntraprofessional team members^26,30,31,50,52^
	oTeamwork^26,28,33,45,47,67^
	oSupervisor relationship or management support^41,47^
	oOrganizational mental health support^57^
4. Importance of support services, society support, and scientific information	oWell-Being Teams^8^
	oResilience in stressful events program and employee assistance program^33^
	oProfessional organizations^31^
	oEthics consultation^26^
	oPolicies^26,52^
	oExternal societal supports from governments and community^31,41,50,67^
	oHigher salaries, career development, giving honorary title, professional recognition, and funding for more medical protective supplies^41^
	oSocial media used to increase people’s sensitivity to the disease, provide preventive teaching on the disease, and maintain an active presence of nurses in the community^50^
	oPublished studies, guidelines, and professional society information to distribute the most up to date and scientifically sound information^31,41,52^
5. Reaffirmation and passion in nursing identity and profession	oGreater sense of professional identity^45^
	oSense of belonging, mission, and duty^42^
	oFlourishing of professional values such as empathy, humanity, and altruism and a sense of being responsible^50^
	oChanges and reaffirmation of nursing identity^28,42^
	oStrong “civic sense” or social responsibility^28,50^

#### Category i: morally effective care

Some nurses described feeling able to effectively overcome ethical challenges encountered, by providing care that aligned with patients’ values.^26,50^ Abbasinia et al.^50^ outlined ways in which nurses described feeling able to provide effective, humanistic care because they followed their professional code of ethics and maintained their conscience and commitment to the profession and society. Both Abbasinia et al.^50^ and Voultsos et al.^22^ indicated that nurses embraced empathic approaches to the provision of care and described feeling able to fulfill their responsibilities to patients.

However, many of these authors also described how there was often a cost associated with these efforts as they captured nurses’ narratives that demonstrated ways in which they deprioritized their own safety or well-being to provide morally effective care.^22,26,49,50,55,58^ For example, Abbasinia et al.^50^ provide the following quote: ‘‘Although I have diabetes and feel very tired because of the busy shifts, not only I don’t leave the patient, but I come to the hospital during peak hours to help people’’ (p. 5).

#### Category ii: self-care strategies and resilience

To cope with the multitude of pandemic-related challenges, nurses described drawing upon their resilience,^21,57^ creativity, and flexibility.^21^ Melnikov et al. described resilience as “being able to work long shifts with extra hours in a tough environment while wearing protective gear, and moreover still being ready to take on new work and tasks” (p. 409). Morley et al.^26^ sought to understand if nurses drew upon moral resilience to overcome ethical challenges and found that they drew upon some facets of moral resilience (as defined by Rushton^59^) but not all. In this study, nurses described drawing upon their own personal strengths and values to make “good” decisions and provide “good” care, they problem-solved, described metacognitive methods used to overcome challenges, and sought team support.^26^

Authors commonly identified various self-care and coping strategies described by nurses such as “time-out” and mindful[ness] moments,^57^ exercise, reading, listening to music, prayer/mediation, watching TV/movies,^28,47^ and developing hobbies.^41^ Though, as reported by Chipps et al.,^57^ nurses also reported finding it challenging to find time to engage in self-care activities and use mental wellness resources: “The organization's been great about creating those mental health tools and services. They're there. You just have to take advantage of ‘em. You often say like: ‘I don't have time to deal—look for some things supportive right now. I'll just try to dig through the next thing to do’” (p. 350).

#### Category iii: collegial and team peer support

Many authors described how teams of nurses came together to support one another.^22,26,28,31,33,45,47,57^ Nurses used various sources and approaches to collegial and peer support to try and overcome the ethical challenges encountered during the pandemic.^22,33,47^ Sources of support ranged from peers,^26,28,33,57^ supervisor relationships or management support,^41,47^ intraprofessional team members,^26,30,31,50,52^ and general teamwork.^26,28,33,38,45,47^

#### Category iv: importance of support services, society support, and scientific information

Nurses described using support service and resources to help them overcome ethical challenges, ranging from ethics consultation,^26^ “Well-Being Teams,”^8^ a resilience program,^33^ Employee Assistance Program,^33^ policies^26,52^ published studies, guidelines, professional society information to distribute the most up to date and scientifically sound information,^31,41,52^ social supports,^27,28^ and spiritual supports.^33^ Specific societal supports from the government and community included supportive strategies such as higher salaries, career development, giving honorary titles, professional recognition, and funding for more medical protective supplies.^41^

More broadly still, nurses described how support from society helped to boost their morale as they received recognition for their work.^31,38,41,50^ The increased sense of solidarity enabled them to feel motivated and focused.^38^ In addition, the improved relationship between the nursing profession and society was perceived as strengthening community trust in nursing and improving the public image of nursing.^50^

#### Category v: reaffirmation and passion in nursing identity and profession

For some nurses, the crisis of the pandemic led to the reaffirmation of their sense of professional identity.^28,45^ Authors described how the challenge of the pandemic enabled some nurses to reconnect and fulfill their professional values of empathy, humanity, and altruism,^50^ which renewed their passion, commitment,^28^ and sense of belonging within nursing.^42^

## Discussion

Our review aimed to scope the ethical challenges nurses experienced while caring for patients during the COVID-19 pandemic. One of the most striking findings from this review was the extent to which many ethical challenges were interrelated, and so categories overlapped in significant ways. For example, nurses described having to take on additional responsibility for “non-nursing” tasks (both skilled and non-skilled), due to both the need for infection prevention and the lack of support staff. However, this resulted in diminishing nurses’ ability to spend time on patient care, which then compromised care quality, causing nurses to feel morally distressed, devalued, and exacerbated inequities between healthcare professionals. The interrelated nature of ethical challenges was also due, in part, to the inclusion of many qualitative studies that used various approaches to data analysis which sought to understand complex phenomena in context. In the future, qualitative researchers might consider developing and sharing an international code book to identify and report ethical challenges accurately and consistently, or working in international teams to map themes as they emerge. This would facilitate naming specific ethical challenges, which could then inform specific responses.

Though other published studies describe the ethical challenges nurses faced during the COVID-19 pandemic and the impact on nurses,^3,17^ information relating to how nurses addressed these challenges remains limited with only a few of the included studies capturing nurse’s specific responses. Furthermore, when responses to ethical challenges were described, it was rather vague and general. For example, Abbasinia et al.^50^ described one theme, “flourishing of professional values,” and provided examples of ways in which nurses felt able to provide ethical care during the pandemic, but the authors did not detail how this response met a specific challenge encountered. Similarly, in the sub-category, “morally effective care,” we captured some ways in which nurses had described feeling able to fulfill their obligations to patients.^22,26,50^ Authors indicated that some nurses drew upon codes of ethics, others drew upon their conscience, and some seemed to de-prioritize their own needs, which came at personal cost.^22,26,49,50,55,58^ However, it was not clear which ethical challenges they were responding to when they drew upon these resources.

Many of the other strategies described in the studies were fairly superficial in terms of their responses to ethical challenges and included self-care and peer support. These approaches are important and helpful but are not a replacement for institutional leadership support and expert ethics support and guidance. Few of the papers included in this review mentioned utilizing specific ethics support and ethics consultation services. There were calls in the height of the pandemic for properly resourced clinical ethics support structures.^75^ As we work towards recovery, we need to review the decisions that were made through a critical lens and work towards implementing recommendations, including ethical capacity-building, so that we are better prepared to address these ethical challenges in the future. Furthermore, capturing both the ethical challenge and the related causal response with specificity would enable an understanding of the most effective responses.

This scoping review highlighted the complexity and scope of ethical challenges nurses faced and navigated under sub-optimal conditions during the COVID-19 pandemic. In particular, nurses experienced frequent conflicts when trying to balance their responsibilities to patients with preserving their own safety demonstrating the need to cultivate workplace environments that are ethical, safe, and just in which nurses feel supported to meet (at least) their basic obligations to patients. Nurses described inadequate workplace conditions, and lack of clarity regarding priority setting and resource allocation resulting in experiences of moral distress, indicating a need for better decisional support processes. Nurses had to draw upon their own personal values and conscience to make decisions and described routinely sacrificing their own well-being. These approaches are insufficient and ought not to be regarded as acceptable.

During the pandemic, nurse leaders still had to fight for a seat at the table in various policy-making spaces.^76^ This scoping review identified the need to strengthen ethical leadership in nursing,^5^ including during sub-optimal conditions during global and public health emergencies like the COVID-19 pandemic. A well-established body of literature in nursing shows the importance, pivotal role, and far-reaching impact of robust ethical nursing leadership on patient care.^62,63^ Future research and education ought to be focused on developing effective, ethically knowledgeable leaders who can represent and advocate for the nursing workforce and the unique challenges that nurses encounter.

The COVID-19 pandemic exacerbated health disparities, disproportionately affecting vulnerable populations such as individuals and communities in low- and middle-income countries, racial and ethnic minorities, and people with disabilities.^77^ This review revealed that nurses navigated many ethical challenges related to neglected patient rights, and significant structural and systemic disparities arose due to failures in pandemic preparedness and responses. The COVID-19 pandemic highlighted nurses’ unique role and ethical responsibilities in advocating for vulnerable populations and in navigating cultural differences and structural and systemic inequities. Using ethics as a source of critical consciousness that is embedded in global and public health emergency responses must be the lens through which decisions and actions are taken, as it allows nurses and nurse leaders to draw attention to structural and systematic health inequities and inequalities and unexamined norms in pandemic responses.^65^ Nurse leaders with greater knowledge of ethical theory and skills in ethical analysis can contribute more effectively to ethical decision-making, helping to ensure that responses to crises do not deepen existing inequities and disparities. We identified issues related to failures in enforcing fair allocation of scarce resources, transparency in communication, accountability, and shared responsibility.^30,42^ Capacity-building efforts in nursing to educate and foster ethical leadership competencies and capacities among nurses in the recovery of the COVID-19 pandemic should be a priority.^64^

The review revealed the imperative for future preparedness for global and public health emergencies and disasters, but especially preparedness informed by ethics and ethical values.^5,29,30,37^ Ethical preparedness encompasses equipping and supporting nurses, and other healthcare professionals, with competencies and strategies to name, frame, and respond to ethical challenges effectively so that they can engage in local decision-making within their work environment, and in broader policy-making forums. Nurses and nurse leaders would benefit from ethics training that is focused on responses to global and public health disasters and emergencies. Healthcare systems need to provide adequate resources and support to protect patient and nurses’ safety with access to surplus equipment to ensure access to PPE. In addition, ethical preparedness requires the creation of workplace environments that foster an ethical culture and climate in which nurses feel empowered to reflect and discuss ethical challenges, advocate for moral health and well-being without fear of professional repercussions, and developed a sense of moral agency and moral clarity, even in crises.^60^ We recognize that realizing this vision is challenging as it will require navigating individual, organizational, environmental, and systemic barriers.^61^

We propose the following six strategies and priorities for ethical pandemic preparedness.

i. Engage nurses, healthcare professionals, leaders, policymakers, professional regulators, and community-level stakeholders in anticipatory planning to develop a comprehensive set of contextualized ethical values and priorities to inform actionable normative recommendations for implementation to guide a just and equitable response to pandemic and disaster scenarios (i.e., triage, rationing, and allocation of resource, surveillance, and monitoring).
ii. Proactively engage vulnerable communities to gain a contextualized understanding of their particular needs, priorities, culture, values, and readiness.^68–71^
iii. Engage in strategic workforce planning and forecasting to identify gaps in nursing workforce preparedness. Compile readily accessible nursing workforce data to inform pandemic and disaster human resource decision-making.
iv. Strengthen and build capacity for a well-trained and supported nursing workforce equipped with practical tools for pandemic and disaster (re)deployment and responses.
v. Engage in educational outreach initiatives and partnerships to incorporate core ethics competencies of emergency preparedness in nursing curricula in culturally and ethically responsible and responsive ways.^70,72,73^ Increase nurses’ ethical readiness and confidence to cultivate ethical knowledge, moral resilience, and mitigate moral distress.^74,29^ In particular, a focus on strengthening nurse leaders’ skills and knowledge in ethics is recommended in local, national, and international fora.
vi. Commit adequate funding for research and scholarship in ethics to inform the development of ethically sound and tested resource allocation guidance.

Global and public health emergencies like the COVID-19 pandemic and its global implications highlighted the imperative for ethical solidarity, human dignity, relationality, and required cross-cultural collaboration and sensitivity. Future practice, research, and policy efforts must focus on ethical preparedness through proactive strategic resource and workforce planning, and clear and actionable consensus-based normative recommendations and policies to guide nurses’ ethical decisions and practice. Through cultivating global partnerships, we can engage in ongoing collective advocacy and action to engage in meaningful action to better address future ethical challenges effectively and in culturally responsible, responsive, and nuanced ways.

## Limitations

Though we attempted to capture contextual features related to ethical challenges, most authors did not report them as specifically related to the broader healthcare systems in which they operated. This review does not capture variability in national healthcare systems, institutional responses, and regional COVID-19 policies. In line with scoping review methods, we limited our interpretation and categorized findings using the unique and particular names assigned by study authors, leading to some overlap between categories. In addition to the identified limitations, this scoping review has strengths. The rigorous review steps employed throughout the scoping review phases, including using multiple investigator triangulation (two reviewers and a third member mitigating any disagreements), helped to strengthen the credibility and trustworthiness of the results.

## Conclusion

The scoping review revealed that the literature on the ethical challenges nurses experienced while caring for patients during the COVID-19 pandemic are numerous and interrelated. Many of the reviewed studies used a qualitative methodology to explore and describe the phenomenon under investigation. The findings of this scoping reveal a significant gap in the nursing research literature regarding specific responses to address the ethical challenges identified. Our results are important and informative for nurses in education, research, and policy. The findings can inform future pandemic preparedness planning, research agendas, and the development and design of ethics education and training curricula, policy formulation to facilitate ethical leadership, and practice environments that optimize nurse safety.
